# A New Approach to Surgical Management of Tibial Plateau Fractures

**DOI:** 10.3390/jcm9030626

**Published:** 2020-02-26

**Authors:** Stuart A. Callary, Claire F. Jones, Karim Kantar, Heleen Du Toit, Markus P. Baker, Dominic Thewlis, Gerald J. Atkins, Lucian B. Solomon

**Affiliations:** 1 Department of Orthopaedics and Trauma, Royal Adelaide Hospital, Adelaide, SA 5000, Australia; karimkantar@gmail.com (K.K.); bogdan.solomon@sa.gov.au (L.B.S.); 2 Centre for Orthopaedic and Trauma Research, The University of Adelaide, Adelaide, SA 5000, Australia; claire.jones@adelaide.edu.au (C.F.J.); linkydt@hotmail.com (H.D.T.); markusbaker@hotmail.com (M.P.B.); dominic.thewlis@adelaide.edu.au (D.T.); gerald.atkins@adelaide.edu.au (G.J.A.); 3 School of Mechanical Engineering, The University of Adelaide, Adelaide, SA 5000, Australia; 4 Epsom Hospital, Dorking Road, Epsom, Surrey KT18 7EG, UK

**Keywords:** tibial plateau fracture, infection, surgical approach, angiosome

## Abstract

Tibial plateau fractures (TPFs) are challenging, requiring complex open reduction and internal fixation (ORIF) and are often associated with complications including surgical site infections (SSIs). In 2007, we introduced a novel management protocol to treat TPFs which consisted of an angiosome- or perforator-sparing (APS) anterolateral approach followed by unrestricted weight bearing and range of motion. The primary aim of this retrospective study was to investigate complication rates and patient outcomes associated with our new management protocol. In total, 79 TPFs treated between 2004 and 2007 through a classic anterolateral surgical approach formed the “Classic Group”; while 66 TPFS treated between 2007 and 2013 formed the “APS Group”. Fracture reduction, maintenance of reduction and patient-reported outcomes were assessed. There was a clinically important improvement in the infection incidence with the APS (1.5%) versus the Classic technique (7.6%) (1/66 versus 2/79 for superficial infections; 0/66 versus 4/79 for deep infections). Despite a more aggressive rehabilitation, there was no difference in the fracture reduction over time or the functional outcomes between both groups (*p* > 0.05). The APS anterolateral approach improved the rate of SSIs after TPFs without compromising fracture reduction and stabilisation. We continue to use this new management approach and early unrestricted weight bearing when treating amenable TPFs.

## 1. Introduction

Tibial plateau fractures (TPFs) are regarded as challenging fractures to reduce and stabilise, typically requiring complex open reduction and internal fixation (ORIF) commonly performed through an anterolateral approach [1]. Loss of fracture reduction, knee stiffness and prolonged rehabilitation are common problems. However, postoperative surgical site infections (SSI) are of particular concern. TPFs have a higher rate of SSIs compared to most other fracture types and are in themselves considered an independent risk factor for SSIs [2]. Other non-modifiable factors are known to compound the risk of SSIs after a TPF including open fractures, compartment syndrome with emergent fasciotomy, male gender, smoking and increased American Society of Anesthesiologists (ASA) Physical Status Classification [3,4]. All complications extend treatment duration, negatively affect patient outcomes, have the potential to reduce patient’s quality of life and can increase health care costs by >300% [5]. Clinical practice changes that can reduce the rate and severity of complications and in particular SSIs are important for both the patient and health care systems.

Recently, it was proposed that an angiosome-sparing anterolateral surgical approach to treat TPFs is possible and that such an approach may help reduce the risk of SSIs [6]. An angiosome is a composite tissue block of bone, muscle, fascia and skin which are linked three dimensionally by anastomotic arteries from one source vessel [7]. Dissection through any angiosome is known to devascularise the angiosome in part or entirely [8]. Traditionally, anterolateral approaches to treat TPFs are performed through curvilinear incisions over the anterior tibial angiosome. Such approaches can damage skin perforators and involve elevation of the tibialis anterior muscle [9], thus dissecting through this angiosome at multiple levels. When an angiosome-sparing approach is not possible, a perforator-sparing approach could be used as the next level of soft tissue protection. We suggest that using an Angiosome of Perforator-Sparing (APS) approach may allow improved outcomes and fracture fixation [6]. Firstly, by enabling the surgeon to position the lateral plate more anteriorly or posteriorly on the lateral tibial condyle, fixation of the lateral condyle is maximised. Secondly, by offering the ability to combine this approach safely with any combination of secondary approaches to the proximal tibia as dictated by the fracture pattern, more plates and screws can be applied to the proximal tibia without further compromising blood supply or increasing complications.

The primary aim of this study was to investigate patient outcomes and postoperative complications following the introduction of a new management protocol for the treatment of TPFs. The proposed new management protocol includes the use of an APS surgical approach, early weight bearing as tolerated after surgery, and unrestricted postoperative range of motion.

## 2. Materials and Methods

Patients treated at our institution for a TPF between January 2004 and May 2013 by one surgeon (LBS) were included in this study. Inclusion criteria for this study were: an acute TPF (<six weeks old), surgical approach including an anterolateral approach, no active leg infection at the time of surgery, and a minimum of 2 years follow up. Exclusion criteria were: patients residing remotely and not able to attend regular follow ups. Depending on the type of anterolateral approach, the patients were separated into an APS sparing Group [6] (September 2007–May 2013), and Classic Group using an anterolateral approach [9] (January 2004–September 2007). Our project received ethics approval from our respective university and hospital committees. This project was a retrospective analysis of data collected prospectively.

### 2.1. Angiosome- or Perforator-Sparing Surgical Technique

**Figure 1:**
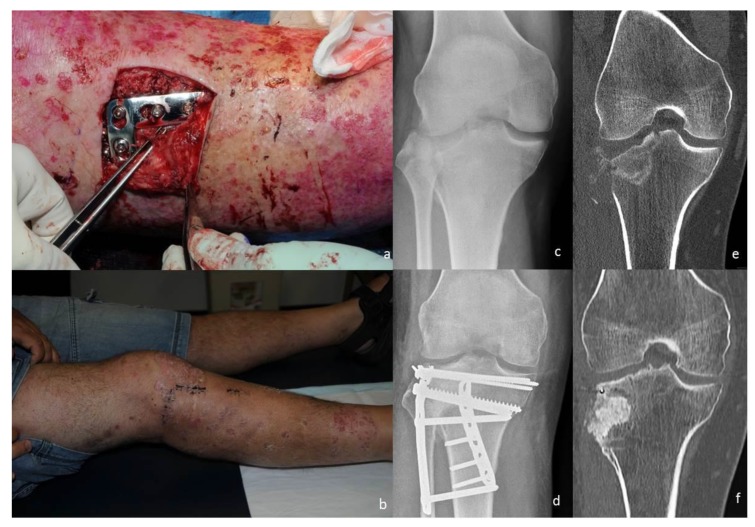
A tibial plateau fracture in a 40-year-old male with extensive florid psoriasis. The patient was managed surgically through a combined angiosome- or perforator-sparing anterolateral approach and a posteromedial approach on day 2 postinjury. (**a**) Intraoperative image, demonstrating the angiosome-sparing anterolateral approach and the psoriasis lesions around it. The plate is positioned while preserving the perforating vessel (elevated by the forceps). (**b**) At 2 weeks postoperative review, the wound is healed. (**c**) Preoperative anterior-posterior radiograph illustrating the fracture. (**d**) Postoperative radiograph showing the reduction and fixation achieved through a combined angiosome-sparing approach and a posteromedial approach. Note the lateral plate sitting on the tibia, as an internally placed external fixator. (**e**) Preoperative coronal computed tomography (CT) image illustrating the fracture. (**f**) Coronal CT image taken after plate and screws were removed 18 months after initial fracture.

**Figure 2:**
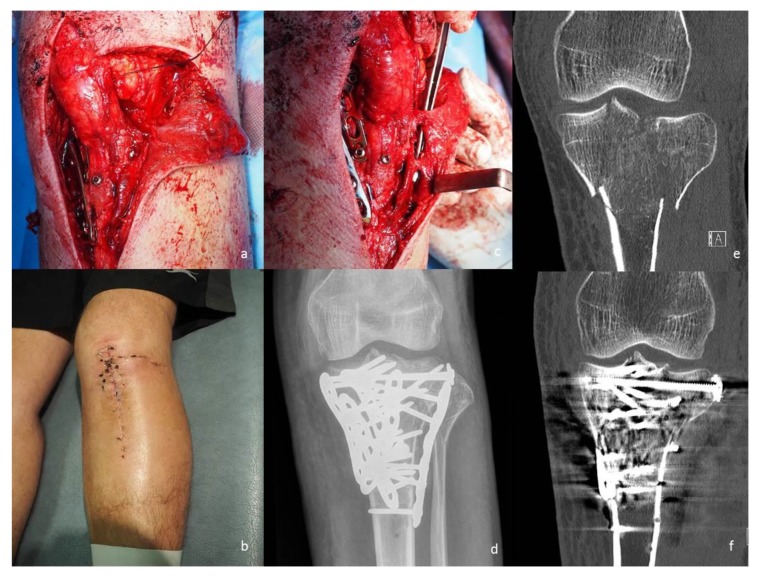
(**a**) The exposure used for a combined perforator-sparing anterolateral and anteromedial approach in a case treated through a combination of anterolateral, anteromedial and posteromedial approaches. (**b**) Clinical image at 2 weeks follow up, demonstrating the healed skin incisions used for the combined anterolateral and anteromedial approaches. (**c**) Note the disposition of the perforator vessels, which in this case precluded placement of a L shape plate on the lateral tibial condyle without kinking of the vessels. To prevent kinking of these vessels, the lateral fixation was done with a straight plate inserted under the anterior margin of tibialis anterior through a perforator-sparing approach. Note the two anteromedial plates in place. (**d**) Postoperative AP radiograph of the knee, demonstrating an ‘aggressive’ fixation, used with five plates and screws. (**e**) Preoperative coronal CT image illustrating the fracture. (**f**) Postoperative coronal CT image.

The skin incision was performed between the knee angiosomes along the anterosuperior margin of the anterior tibial angiosome [7] and thus involved a vertical (midline) and a horizontal arm (along the lateral joint line of the knee) at 90° to each other. A full thickness flap was mobilised from the crural fascia and the skin perforators closest to the skin incision were exposed and protected (Figure 1). If Gerdy’s tubercle was fractured, it was mobilised proximally on the iliotibial band to expose and reduce the fracture through a sub-meniscal dissection plane. Otherwise, the knee joint was exposed through a lateral para patellar arthrotomy with, or without, a second arthrotomy between the iliotibial band and the lateral collateral ligament, as previously described [6]. If a split component of the fracture was present anterior to Gerdy’s tubercle, this was opened using laminar spreaders to expose and reduce the depressed fracture fragments. If no split fracture component was present anterior to Gerdy’s tubercle an osteotomy of the anterior margin of the plateau was performed at this level. At no time during this phase of fracture reduction was any of the tibialis anterior exposed or mobilised. Lateral condyle fracture fixation was performed with a raft of subchondral screws (Synthes, Paoli, PA, USA) and a locking plate/screw construct (Synthes, Paoli, PA, USA). If the anatomical position of the perforators did not interfere with the position of a locking plate placed on the tibialis anterior fascia this was done as per a true angiosome-sparing approach; an “L” shaped buttress locking plate (Synthes, Paoli, PA, USA) was straightened to remove its anatomical contour [6] and used as an internally placed ‘external fixator’ over the fascia of tibialis anterior (Figure 1). If, however, the anatomical position of the perforators precluded the positioning of the plate over the fascia without kinking or lacerating some of the perforators a compromise from the true angiosome-sparing approach was made. In these patients (14 in total), after fracture reduction, the tibialis anterior was mobilised subperiosteally from the anterior tibial margin for an area equivalent with the area of a small fragment plate next to the anterior margin of the tibia such that the plate could be applied without interference with the skin perforators (Figure 2), a perforator-sparing approach.

When a true angiosome-sparing approach was performed, the lateral plate was placed more anterior or posterior on the rim of the lateral tibial condyle such that the plate best stabilises the split component of the lateral tibial condyle. When a true angiosome-sparing approach was not possible, the plate was placed subperiosteally along the anterior margin of the tibia to minimize the dissection to tibialis anterior.

### 2.2. Rehabilitation

After surgery patients were encouraged to mobilise as early as possible, with no restriction on weight bearing and knee range of motion. Patients were encouraged to use crutches for the first 6 postoperative weeks.

### 2.3. Outcome Measures

Fractures were classified according to the Schatzker [10] and OTA [11] classifications. Patient reviews were conducted daily as inpatients and then at 2, 6, 12 and 26 weeks and 1 to 2, and 3 to 5 years after injury.

All complications were recorded. Infections were defined according to the criteria of the Centre for Disease Control and Prevention as superficial or deep, and diagnosed based according to the Hospital Infection Control Practices Advisory Committee [12].

Radiographic assessment of fracture reduction (after surgery) and maintenance of fracture reduction (at last follow up) was done on plain radiographs and on computed tomography (CT) scans when available using criteria described by Rasmussen: fracture depression, tibial condylar width, articular step and condylar tilt [13]. Radiographs were available for all patients for assessment. At our institution, preoperative CT scans were taken routinely and postoperatively, and a CT is only requested when quality of reduction is unclear on radiographs.

Functional outcome was assessed via the patient-reported Lysholm scores [14]. This was determined after fracture healing, at 1 to 2 years after surgery, and then at 3 to 5 years after surgery.

### 2.4. Statistical Methods

All statistical analyses were performed using SPSS (v 22.0.0.0, IBM Corporation, Armonk, NY, USA). Two-tailed Pearson chi-squared tests or Fishers exact tests (where one or more cells had a count <5) were performed to determine whether there was an association between treatment group and each of the categorical parameters. Mann–Whitney U tests were performed to determine whether there was an association between treatment group and each of the ordinal variables. Univariate Analysis of Variance (ANOVA), followed by Tukey HSD tests for multiple comparisons if required, was performed for each continuous variable to determine whether there was a difference between groups. Statistical significance was defined by *p* < 0.05.

## 3. Results

During the study period, 216 patients were treated surgically in our hospital for a TPF. Of these, 143 patients (with 145 TPFs) met our inclusion criteria. The APS Group comprised 66 TPFs, while the Classic Group comprised 79 TPFs. All data were prospectively documented and retrospectively collated. Patients were followed up for a minimum of 2 years. The summary of their demographic data can be found in Table 1.

### 3.1. Radiographic Outcomes

Maintenance of fracture reduction across both groups is summarized in Table 2. The mean changes in radiographic parameters over time for the Classic Group and the APS Group respectively were: 0.6 mm+/−2.7 and 0.12 mm+/−1.9 (*p* > 0.05) for lateral step; 0.5 mm+/−5.4 and 2 mm+/−6.7 (*p* > 0.05) for tibial widening; 0°+/−3.3 and −0.2°+/−3.24 (*p* > 0.05) for condylar tilt. The numbers of patients for which adequate radiographs were available for analyses for the Classic Group were: 74 pre-operatively and immediately after surgery and 76 at the last follow up. The numbers of patients for whom radiographs were available for analysis for the APS Group were 63 preoperatively and 66 immediately after surgery and at last follow up.

### 3.2. Patient-Reported Outcomes

The Lysholm scores for the Classic Group and the APS Group at 2 years were 63.5+/−25.6 (N = 26) and 70.3+/−20.9 (N = 45), *p* > 0.05, and 67+/−21.4 (N = 38) and 75.8+/−21.3 (N = 16), *p* > 0.05, at last follow up.

### 3.3. Complications

Five complications occurred in the APS Group: one patient developed a deep vein thrombosis (DVT); two patients developed a postoperative footdrop that resolved completely within six months; one patient developed a deep peroneal nerve neurapraxia which self-resolved; and one patient developed a superficial infection, which resolved with antibiotics alone. No patient developed a deep infection.

Six complications occurred in the Classic Group: two patients developed a superficial infection treated with antibiotics and four patients developed a deep infection which required surgical debridement’, subsequent removal of metalwork and prolonged antibiotics.

### 3.4. Re-Operations

Four patients in the APS group underwent further surgery within the study period for knee pain and instability. All had their metalwork removed and an arthroscopy performed. Of these, one patient had a subsequent ACL reconstruction, two patients had an arthroscopic meniscal debridement and one patient required an open meniscectomy for a bucket handle tear.

Two patients in the Classic Group required a diagnostic arthroscopy with removal of metal. Two patients went on to receive a total knee replacement for posttraumatic arthritis at two years after their initial ORIF.

## 4. Discussion

This study investigated the complication rates and outcomes associated with the introduction of a new management protocol for the treatment of TPFs and compared these results with those of patients treated before the implementation of these changes. Our prior studies have suggested that an angiosome-sparing anterolateral approach is possible and that such an approach may reduce the SSI risk after TPFs [14]. We have also used accurate radiographic measurements in a small cohort to demonstrate that TPF fragments do not undergo significant displacements under load in the first 6 postoperative weeks [15]. This allowed us to change our postoperative instructions from non or partial weight bearing in the first six postoperative weeks to unrestricted postoperative weight bearing from the early postoperative period.

This study suggests that the new APS surgical approach, combined with early weight bearing and range of motion, can provide non-inferior clinical and radiological results in a difficult fracture group. There appears to be a clinically important improvement in the infection incidence with the new management technique (1/66 versus 2/79 for superficial and 0/66 versus 4/79 for deep infections).

A clinically important finding of our study was that the rate of deep SSIs was zero using the new APS approach despite these fractures being treated with more plates, which is traditionally associated with increased infections [16]. The APS approach was also found to not negatively affect the ability to reduce and fix the fracture, nor the short-term follow-up functional outcomes. Similarly, the more aggressive mobilisation of patients did not translate to increases in delayed wound healing but seemingly the opposite. Preservation of skin perforators during surgery is known to be important in reducing the rates of SSIs after ORIF of tibial pilon fractures, and we propose the same benefits for TPFs [17]. The current study complements the results of a previous report on the treatment of later TPFs through an angiosome-sparing approach [6], and those reporting on tibial pilon fractures [17] to cement the important role of preserving the regional blood supply in decreasing the rate of SSIs. Whilst we have not specifically looked for any advantage of early weight bearing after surgery in these patients, in general, the deleterious effects of prolonged non-weight bearing are well known, and we propose that this new method of management will likely decrease these effects.

The patients in the APS Group actually had improved fracture reduction. One possible explanation for this is related to the wider exposure of the articular surface of the lateral tibial plateau that can be achieved through the horizontal arm of the incision, located at the level of the joint line. As previously reported [6] this incision allows exposure of the lateral tibial plateau through two windows: one anterior, between the patellar tendon and the iliotibial band; and one posterior, between the iliotibial band and lateral collateral ligament. One thing of potential concern with the APS Group is the unexplained rate of temporary nerve damage compared with the Classic Group. We have no explanation for this increase, as the dissection is always away from the common peroneal nerve. Although all postoperative nerve symptoms resolved completely, this matter requires ongoing attention.

This retrospective study has a number of limitations. First, the number of patients was relatively small, but this is typical of a single-centre study; due to low incidence of infection, larger cohorts are required to confirm the reduction in infections observed in our study. Second, whilst being a retrospective study of prospectively collected data, there was no randomisation with a control group and thus interpretation of the results is limited. Third, CT measurements of fracture reduction are more accurate than plain radiographs. However, CT scans are not taken routinely in our institution due to additional radiation exposure. Fourth, it could be argued that the improved results are not the result of an improved technique, but of the surgeon responsible for the surgery getting better at what he does. We believe this is not the case for several reasons; the surgeon had more than 15 years of experience prior to any of these cases, and the total number of cases he treated surgically during the study period actually decreased. Fifth, data were not available for all study participants; not all patients had adequate imaging at all time points and valid Lysholm scores were not available for all patients.

## 5. Conclusions

In conclusion, this retrospective study has demonstrated that preserving the skin perforators over the lateral tibial condyle when performing ORIF of lateral TPFs through an APS approach facilitated adequate fracture reduction. Infections were low in the APS group, with a deep infection rate of zero. Early mobilisation was possible, without compromise of fracture fixation, maintenance of fracture reduction or early functional outcomes of these fractures. We found no significant increase in wound complications or deterioration in maintenance of fixation despite the earlier and more aggressive rehabilitation process. We therefore continue to use this approach when treating TPFs amendable to an anterolateral approach and recommend its use as it may be used in other centres to decrease their rates of SSIs after TPF.

## Figures and Tables

**Table 1:** Patient demographic, fracture classification and fixation details for both groups.

	Classic Group	APS Group
Number of TPFs (Patients)	79 (79)	66 (64)
Mean Age (yrs, range)	48 (17–94)	45 (18–96)
Sex (Male:Female)	51:28	41:25
ASA grade (I:II:III:IV:V:unknown)	29:40:6:0:0:4	25:28:11:0:0:2
Schatzker classification (I:II:III:IV:V:VI:unknown)	0:46:0:1:7:25:0	0:39:1:0:5:20:1
AO classification (B2:B3:C2:C3)	0:52:1:26	1:42:3:19
Number of approaches used in each case ^1^ (single:double:triple)	54:25:0	34:31:1
Number of plates used in each case (1:2:≥3)	59:18:2	37:20:9
Mean admission to discharge (days, SD)	13.71 (SD11.25)	13.03 (SD12.31)
Mean admission to surgery (days, SD)	4.03 (SD3.98)	4.23 (SD3.47)
Mean surgery to discharge (days, SD)	9.68 (SD10.00)	8.80 (SD10.35)
Mean operative duration (hours, SD)	3.60 (SD1.24)	3.59 (SD1.21)

^1^ All patients were treated through an anterolateral approach. In 25 patients in the Classic Group and 31 patients in the APS Group, this was combined with a posteromedial approach. For one patient in the APS group, the anterolateral approach was combined with a posterolateral and an anteromedial approach. AO: Arbeitsgemeinschaft für Osteosynthesefragen/The Association of the Study of Internal Fixation.

**Table 2:** Fracture displacement parameters before surgery, after open reduction and after fracture healing respectively (at last follow up) for both groups.

	Preop		Immediate Postop		Last Follow Up	
	**Tibial Condyle Widening [mm, range]**	**Lateral Articular Step [mm, range]**	**Tibial Condyle Widening [mm, range]**	**Lateral Articular Step [mm, range]**	**Tibial Condyle Widening [mm, range]**	**Lateral Articular Step [mm, range]**
Classic Group	12.5 [1.5–31.2]	12 [0–43]	6 [−5–18.6]	2.4 ^1^ [0–12.7]	6.2 [−2.3–17.5]	2.8 ^2^ [0–13]
APS Group	9.5 [−1.5–34.5]	11 [0–53]	4.3 [−4–12.7]	0.4 ^1^ [0–5]	5.3 [−3–10.7]	0.4 ^2^ [0–5]
	**Condylar Tilt [degrees, range]**	**Condylar Angulation [degrees, range]**	**Condylar Tilt [degrees, range]**	**Condylar Angulation [degrees, range]**	**Condylar Tilt [degrees, range]**	**Condylar Angulation [degrees, range]**
Classic Group	83 [65–104]	88 [72–103]	84 [75–98]	88 [81–97]	83 [75–91]	88 [81–99]
APS Group	82 [57–107]	85 [69–100]	83 [68–94]	87 [77.6–92.9]	83 [73–93]	86.5 [76–98]

^1^*p* < 0.05; ^2^ *p* < 0.05.
